# Direct Exploration of the Role of the Ventral Anterior Temporal Lobe in Semantic Memory: Cortical Stimulation and Local Field Potential Evidence From Subdural Grid Electrodes

**DOI:** 10.1093/cercor/bhu262

**Published:** 2014-12-09

**Authors:** Akihiro Shimotake, Riki Matsumoto, Taiji Ueno, Takeharu Kunieda, Satoru Saito, Paul Hoffman, Takayuki Kikuchi, Hidenao Fukuyama, Susumu Miyamoto, Ryosuke Takahashi, Akio Ikeda, Matthew A. Lambon Ralph

**Affiliations:** 1 Department of Neurology; 2 Department of Epilepsy, Movement Disorders and Physiology; 3 Department of Neurosurgery; 4 Human Brain Research Center, Kyoto University Graduate School of Medicine, Kyoto, Japan; 5 Neuroscience and Aphasia Research Unit (NARU), School of Psychological Sciences, University of Manchester, Manchester, UK; 6 Department of Cognitive Psychology in Education, Graduate School of Education, Kyoto University, Kyoto, Japan

**Keywords:** anterior fusiform, basal temporal language area, semantic memory, subdural electrodes, ventral anterior temporal lobe

## Abstract

Semantic memory is a crucial higher cortical function that codes the meaning of objects and words, and when impaired after neurological damage, patients are left with significant disability. Investigations of semantic dementia have implicated the anterior temporal lobe (ATL) region, in general, as crucial for multimodal semantic memory. The potentially crucial role of the ventral ATL subregion has been emphasized by recent functional neuroimaging studies, but the necessity of this precise area has not been selectively tested. The implantation of subdural electrode grids over this subregion, for the presurgical assessment of patients with partial epilepsy or brain tumor, offers the dual yet rare opportunities to record cortical local field potentials while participants complete semantic tasks and to stimulate the functionally identified regions in the same participants to evaluate the necessity of these areas in semantic processing. Across 6 patients, and utilizing a variety of semantic assessments, we evaluated and confirmed that the anterior fusiform/inferior temporal gyrus is crucial in multimodal, receptive, and expressive, semantic processing.

## Introduction

Semantic memory or conceptual knowledge is a crucial higher cognitive function. It allows us to code and retrieve the meaning of words, objects, sounds, faces, etc., which has been acquired across a lifetime of verbal and nonverbal experience. Additionally and perhaps reflecting its core essence, semantic memory allows us to generate coherent concepts which license generalizations based on meaning rather than surface similarities (Rosch 1975; Smith and Medin 1981; Wittgenstein 2001; Lambon Ralph et al. 2010). The formation, coding, and correct use of semantic knowledge involves a widely distributed network of cortical regions (Jefferies and Lambon Ralph 2006; Patterson et al. 2007; Binder and Desai 2011; Lambon Ralph 2014). Recent evidence from clinical and cognitive neuroscience (Binney et al. 2010; Mion et al. 2010; Peelen and Caramazza 2012) indicates that, among these areas, regions within the anterior temporal lobe (ATL, bilaterally) are crucial in the representation of coherent concepts (Lambon Ralph et al. 2010), consistent with the functional and structural convergence of information sources that peaks in this area (Morán et al. 1987; Visser and Lambon Ralph 2011; Binney et al. 2012).

The principal evidence for the importance of the ATL in semantic representation arose from the study of semantic dementia (SD), in which a selective pan-modal, pan-category semantic deficit arises from atrophy focused on the anterior temporal region, bilaterally (Patterson et al. 2007). The affected area is, however, a relatively large cortical region that increases as the disease progresses and contains various cytoarchitectural subdivisions (Ding et al. 2009; Rohrer et al. 2009). A crucial question, therefore, is—which areas within the ATL are crucial for semantic processing and how do they vary? Early positron emission tomography (PET) functional neuroimaging studies (which unlike conventional gradient echo planar imaging functional MRI (EPI fMRI) can probe all ATL subregions successfully, see below) indicated that multiple areas within the ATL activate to both verbal and nonverbal semantic tasks (Vandenberghe et al. 1996) as well as to spoken and written word comprehension tasks (Sharp et al. 2004). Functional neuroimaging utilizing standard gradient EPI fMRI has demonstrated that polar and superior ATL regions are important for processing social concepts (Zahn et al. 2007; Ross and Olson 2010), while anterior superior temporal gyrus/sulcus (STG/STS) appears to be important for processing the meaning of auditory sentences (Scott et al. 2000; Vandenberghe et al. 2002), individual auditory words, or nonverbal stimuli (Griffiths and Warren 2004; Visser and Lambon Ralph 2011).

The current investigation was concerned with the role that ventral aspects of the ATL might play semantic memory. This question was motivated not only on theoretical, but also clinical, grounds given that this region is often included in resection for temporal lobe epilepsy, unless a subtemporal procedure for hippocampectomy is used (Mikuni et al. 2006). In comparison with other cortical regions (e.g., prefrontal and temporoparietal regions), there is much less information on the role that the ventral ATL might play in semantic cognition. This is most probably because the common sources of neuroscience data on language and semantics are much less likely to “sample” this region. For example, middle cerebral artery (MCA) stroke (a primary source of information that underpins models of language and aphasia) typically does not damage the inferior-to-middle aspects of the ATL, because it is in the posterior cerebral artery-MCA watershed territory (Phan et al. 2005, 2009). The same regions are often missing from functional neuroimaging studies due to a variety of technical reasons, including magnetic field inhomogeneities (for standard gradient EPI fMRI) and limited field of view (Devlin et al. 2000; Visser et al. 2010). Likewise, repetitive transcranial magnetic stimulation (rTMS) has been used to demonstrate the necessity of left and right lateral ATL areas to multimodal semantic processing in neurologically intact participants (Pobric et al. 2007, 2010a; Lambon Ralph et al. 2009), but, due to its neuroanatomical location, it is impossible to stimulate the ventral ATL directly using TMS.

**Figure 1.**
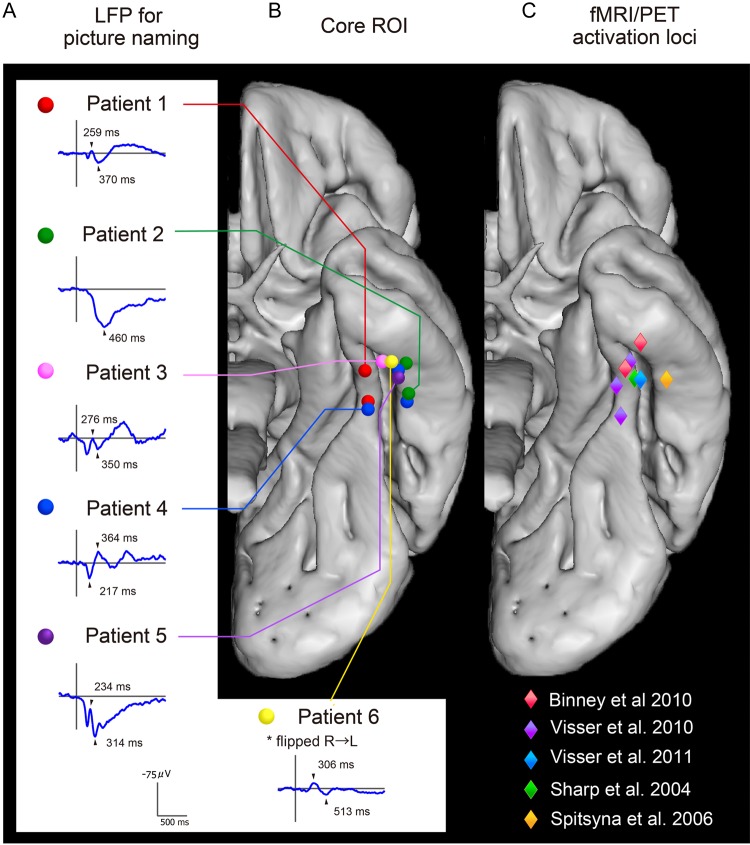
Summaries of the strong convergence of results from the LFPs (*A*—Investigation 1), direct stimulation studies (*B*—Investigations 2–4), and previous functional neuroimaging investigations of verbal and nonverbal semantic processing (peak activations are plotted in *C*). A critical area within the vATL was located around the anterior fusiform gyrus (3.8–5.2 cm from the temporal pole). Representative LFP waveforms (average waveforms from all of the naming session) are shown for each patient (*A*) taken from the same vATL region as that identified in the direct stimulation Investigations 2–4 (*B*) and aligning with the peaks from semantic functional neuroimaging studies (Spitsyna et al. 2006; Binney et al. 2010; Visser et al. 2011) (*C*). The LFP waveform consisted of a negative activity with a peak latency of 230–300 (Patients 1, 3, and 5) and the subsequent positive/negative activity peaking at 310–450 ms (1–5). Patient 6 showed a negative activity with a peak latency around 300 ms, similar to left cases.

There are some strong hints in the literature that the ventral ATL might play an important role in multimodal semantic representation. Although this region is primarily considered to be the apex of the ventral visual stream (Albright 2012), it is becoming increasingly apparent from connectivity studies and functional neuroimaging that this area is much more transmodal in character. First, not only is the ventral ATL (vATL) connected to primary visual areas, but it is also connected to other temporal, limbic, and frontal regions [as shown in: primate injection-based tractography (Morán et al. 1987); human white-matter tractography (e.g., Binney et al. 2012); human resting-state fMRI connectivity studies (e.g., Pascual et al. 2015); and human cortico-cortical connectivity (e.g., Matsumoto et al. 2004)]. Secondly, recent fMRI studies, designed to minimize the technical and methodological issues associated with successful imaging of this region, have demonstrated graded variation of semantic function across the ATL reflecting the pattern of connectivity to remote modality-specific association cortices (Visser and Lambon Ralph 2011; Binney et al. 2012) and the coding of semantic category structure (Peelen and Caramazza 2012). Among other regions [for reviews, see Binder et al. (2009); Visser et al. (2010); Noonan et al. (2013)], the ventral ATL (centered on the anterior fusiform/inferior temporal gyrus, ITG: Fig. 1) is activated, irrespective of variations in task or modality—consistent with previous PET-based functional neuroimaging studies (Vandenberghe et al. 1996; Sharp et al. 2004) and raising the possibility that this area is the center point of a transmodal semantic hub (Binney et al. 2010; Visser and Lambon Ralph 2011). Selective investigation of the vATL is needed for at least 2 reasons: (1) Functional neuroimaging generates important hypotheses about the contribution of specific brain regions, but activation by itself does not demonstrate the necessity of those areas (Price and Friston 2002); (2) while the ventral ATL does exhibit early and disproportionate damage (Galton et al. 2001) and hypometabolism in SD (Mion et al. 2010), the patients' atrophy is not isolated to this specific region (Rohrer et al. 2009).

The current study directly tested the contribution of the vATL region to semantic processing through the implantation of subdural electrode grids, for the presurgical evaluation of patients with partial epilepsy and brain tumor. Unlike functional neuroimaging or TMS alone, this method offers dual unique opportunities: by placing a grid over a region of interest (ROI), it is possible not only to evaluate the functionally related cortical activity directly (evoked local field potentials, LFPs), but also to induce temporary disruption of the function at that site, within the same participant. This is not, of course, the first study ever to have used this method to probe the ventral ATL. In clinical neurosurgery, the same region has been regarded as an important language area—the basal temporal language area (BTLA)—ever since electrical stimulation of this cortical region was found to impair reading and naming (Lüders et al. 1986, 1991), and preservation of this area and its fiber pathway resulted in the better verbal memory outcome after neurosurgery (Mikuni et al. 2006). Implanted grid electrodes were used in another seminal study to measure LFPs in posterior versus anterior ventral temporal areas (Nobre et al. 1994). Consistent with Lüders et al.'s stimulation results, Nobre et al. observed evoked potentials to both verbal and nonverbal visual stimuli (letter strings, written words, and faces) in the ventral temporal areas. Importantly, Nobre et al. also found that the anterior ventral temporal regions and not the posterior areas were sensitive to various semantic manipulations. This result is consistent with the key hypothesis to be tested further in this study and also suggests that the language-related processing associated with the ventral ATL/BTLA might reflect the role of semantic representations in bringing meaning to both verbal and nonverbal stimuli.

Across a series of investigations, therefore, we explored semantic processing of the vATL through the implantation of grid electrodes. The study adopted 3 key methodological approaches. First, we collected both cortical stimulation data and cortical-evoked responses in the same participants, so that we could directly compare functionally related LFPs and test if these same functions were disrupted by transient stimulation (i.e., mirror the direct comparison of fMRI and TMS that has been utilized to probe the function of other cortical regions, including the lateral ATL). Secondly, to license direct comparability with the previous neuropsychological and neuroimaging studies reviewed above, we utilized the same assessment materials and experimental methods leading not only to novel insights about the neural basis of semantic memory, but also to new clinical stimulation methods. Finally, we should note that, given the rarity of the clinical procedure and the limited assessment time with each patient, it was impossible to conduct the large experimental test battery with all patients. Instead, we conducted a core stimulation and evoked potential protocol (Investigations 1 and 2) with all patients and supplemented these with more in-depth investigations across pairs of cases to build up a more complete picture of the nature and extent of semantic processing in the ventral ATL.

## Experimental Procedures

### Participants

We recruited 6 patients with either intractable partial epilepsy (5 patients) or brain tumor (1 patient) who underwent subdural electrode implantation in the left (Patients 1–5) or right (Patient 6) hemisphere for presurgical evaluation. The subdural electrodes were constructed of platinum with an interelectrode distance of 1 cm and a recording diameter of 2.3 mm (ADTECH, WI, USA). The patients' demographics are summarized in Table 1. All patients except for Patient 5 had complex partial seizures with their onset at more than 10 years of age. Hippocampal sclerosis was observed in Patients 2, 4, and 6. Wada test revealed language dominancy in the left hemisphere in Patients 1, 3, 4, 5, and 6, and bilateral in Patient 2 (Table 1). All patients except for Patient 6 had normal language function as assessed by the Japanese version of the Western Aphasia Battery (WAB). Electrocorticographic recording with subdural electrodes revealed the seizure onset zone to be outside the anterior fusiform region in all patients with epilepsy (Patients 1–4 and 6). Intelligence and memory function ranged from mild to moderate impairment to normal except moderate memory impairment in Patients 3 and 6. In addition, we performed the Japanese version of picture naming and written word-picture matching, adapted from the Cambridge 64-item semantic battery to assess semantic cognition (Bozeat et al. 2000) both before (without stimulation) and during (with stimulation) surgery (see below). All patients had good performance (≥90%) before stimulation. Accuracy on additional more stringent semantic tasks [picture–picture semantic association and synonym judgment (Bozeat et al. 2000; Jefferies et al. 2009)] was within the normal range (≥90%) in Patients 2–5. Despite low memory function, we enrolled Patient 3 in this study because of her good performance on the semantic tasks and WAB. Patient 6, who had a low WAB score, was also enrolled because of relatively good performance on the semantic tasks before implantation (≥80% on naming and association task; ≥90% on word-picture matching and synonym judgment). Although only a limited grid (strip) was inserted in the right vATL for Patient 6, we have included the data in this report given the rare opportunity to probe functioning of the right vATL region, directly.

**Table 1:** Patients' demographics and clinical information

Patient	1	2	3	4	5	6
Age, gender, handedness	22, M, R	29, M, R&L	17, F, R	38, F, R	55, M, R	34, M, L
WAIS-R (VIQ, PIQ, and TIQ)	70, 78, 69	72, 78, 72	67, 76, 69	84, 97, 89	105, 99, 103	55, scale out, 44
WMS-R (Verbal, Visual, General, Attention, and Delayed recall)	99, 64, 87, 91, 82	99, 92, 97, 87, 83	51, <50, <50, 81, 56	75, 111, 83, 62, 53	71, 117, 84, 109, 72	52, <50, <50, 55, <50
WAB	95.6	96	97.2	98.5	98	88
WADA test (Language)	Left	Bilateral	Left	Left	Left	Left
Age of seizure onset	16	10	12	29	55	12
Seizure type	Nonspecific aura → CPS, GTCS	Aura (metamorphosia, epigastric rising sensation) → CPS	Discomfort in throat → CPS	Epigastric rising sensation → CPS	CPS (once)	Aura (déjà vu, piloerection, auditory aura) → CPS
Ictal ECoG onset	aMTG	PHG	PHG	PHG	None	R parietal lobe—pMTG
MRI	L basal frontal cortical dysplasia L anterior temporal arachnoid cyst	L posterior temporal cortical atrophy	L temporal tip arachnoid cyst	L hippocampal atrophy/sclerosis	A low-grade glioma in the L medial temporal lobe	R parietal cerebral atrophy and contusion R hippocampal sclerosis/atrophy
Pathology	FCD type IA	FCD type IA Hippocampal sclerosis^a^	FCD type IB	Hippocampal sclerosis^b^	Diffuse astrocytoma	Traumatic head injury Hippocampal sclerosis^a^
Investigation number	1, 2, 3	1, 2, 3	1, 2	1, 2, 4	1, 2, 4	1

CPS, complex partial seizure; GTCS, generalized tonic clonic seizure; ECoG, electrocorticogram; a/pMTG, anterior/posterior part of the middle temporal gyrus; PHG, parahippocampal gyrus; FCD, focal cortical dysplasia.

^a^Dual pathology.

^b^Diagnosed by clinical findings.

This study was approved by the ethics committee of the Kyoto University Graduate School of Medicine (no. C533), and the patients gave written informed consent.

### Investigations

#### Investigation 1 (LFPs)

##### Materials

One hundred (50 animals and 50 nonliving items, matched for visual-complexity, name frequency, age of acquisition, and familiarity) line drawings were collected (Morrison et al. 1997). All could be named reliably by Japanese adults.

##### Procedure

Electrocorticogram (ECoG) was recorded during the picture naming task with a band-pass filter of 0.016–600 Hz and a sampling rate of 2000 Hz in Patients 1 and 3, and with a band-pass filter of 0.016–300 Hz and a sampling rate of 1000 Hz in Patients 2, 4, 5, and 6. Pictures were presented one at a time, every 5 s, on a PC screen.

Participants were asked to name each picture aloud as quickly and accurately as possible. One session consisted of 100 picture naming trials and 4 sessions were performed to obtain robust responses. The participant's behavior and eye fixation were monitored by video recording. LFPs for correct trials were obtained by offline averaging of the ECoG time-locked to the picture onset with a band-pass filter of 0.016–60 Hz, using in-house MATLAB scripts (version 2010a, Mathworks, Natick, MA, USA). A time window of 1800 ms was set with activity in the 300 ms preceding the picture presentation serving as the baseline for measurement. Average waveforms were computed for the first and second half of the experiment to assess the consistency of the LFPs (shown in Supplementary Fig. 1). After confirming the reproducibility, grand average waveforms (for the whole test session) were computed (Fig. 1).

Previous studies have obtained important evidence about the vATL region by directly comparing results from contrastive neuroscience techniques (e.g., neuropsychology, functional neuroimaging, and TMS: Binney et al. 2010). Accordingly, as a supplementary feature of this study, we wished to compare the grid electrode LFP and stimulation results against previous functional neuroimaging explorations of the vATL's contribution to semantic processing. Large-scale meta-analyses of the semantic neuroimaging literature (e.g., Binder et al. 2009; Visser et al. 2010) have found reliable peak activations in multiple ATL regions, most commonly in superior-to-middle and polar ATL areas, as well as other non-ATL brain regions (reflecting the fact that semantic cognition is supported by a large-scale, distributed neural network). With respect to the ventral surface (the focus on this grid electrode study), peak activations are much less common but not entirely absent. This may reflect, in part, various methodological factors (cf. Visser et al. 2010). Combining across 164 fMRI and PET studies of semantic processing using the ALE (activation likelihood estimation) meta-analysis method, Visser et al. did observe a small area of activation likelihood in the anterior ITG (see Figure 3*A* in; Visser et al. 2010). To illustrate the relationship between functional neuroimaging and the current grid electrode data, we compiled a set of more recent semantic studies that have tried to reduce the signal problems associated with the ventral ATL. These are not an exhaustive list of all semantic functional neuroimaging studies (as included in previous large-scale meta-analyses), but they allow us to compare semantically related peak activations when they do occur in the vATL against the critical grid position in each patient (Fig. 1*C*). To generate Figure 1*C*, we undertook the following steps. Consistent with the critical methodological factors for maximizing ATL-related semantic activations established by Visser et al (2010), studies of semantic processing were selected on the basis of the following criteria: (1) PET or distortion-corrected fMRI, with sufficient field of view to cover the full ATL; (2) semantic performance was contrasted against a “high-level” baseline task; and (3) given that we are interested in testing whether this region is involved in multimodal semantic representation (rather than visual recognition alone), we only selected studies that either presented stimuli in the auditory domain or probed abstract concepts (i.e., we did not select any studies that only probed visually presented, pictured concrete concepts).

#### Investigation 2 (Conventional Functional Cortical Mapping)

##### Apparatus

High-frequency electrical cortical stimulation was performed with subdural electrodes. Repetitive, square-wave electric currents of alternating polarity with a pulse width of 0.3 ms and a frequency of 50 Hz were delivered through a pair of electrodes for 1–5 s (Electrical stimulator SEN-7203, Nihon Kohden, Tokyo, Japan). Details of the methodology for cortical stimulation and the subsequent cortical mapping have been described elsewhere (Matsumoto et al. 2011).

##### Assessment criteria

For mapping the vATL, after confirming the absence of positive (e.g., tonic contraction) and negative (e.g., impairment of rapid alternating movements) tongue motor responses, a series of semantic and language assessments was tested concurrent with the 4- to 5-s period of electrical stimulation at an intensity of 10–15 mA. For the assessment battery shown below (Usui et al. 2003), responses were rated as errors when subjects made either no response (arrest), delay in response (slowing), or an incorrect verbal reply (error) during stimulation (Table 2). We assessed the impaired behaviors as significant when the findings were reproducible in the absence of afterdischarges. In case of frequent afterdischarges, we decreased stimulation intensity to 8 or 9 mA so that stimulation did not induce afterdischarges. In Patient 3, very brief afterdischarges of a few seconds were elicited at some electrodes, even though stimulation intensity was decreased. Since the discharges were so brief and behaviors were clearly impaired, it strongly suggests that the cortices at or very close to the stimulation site were responsible for that particular cortical function (Matsumoto et al. 2011). All sessions were video-recorded and ECoG was recorded simultaneously.

**Table 2:** Summary of conventional 50Hz cortical stimulation and the sites of investigations (Patient 1–5)

Stimulus site		Investigation 1	Investigation 2						Investigation 3	Investigation 4
		Robust LFPs	Paragraph reading	Picture naming	Word-picture matching	Spoken verbal command	Kanji word reading	Kana word reading	Graded stimulation	Synonym LFPs/stimulation
Patient 1										
ITG	A08		○	×	×	××	××	○		
	A13/A18*		○	×	×	○	○	○		
	A03		○	○	×	○	○	○		
	A19		○	○	×	○	○	○		
FG	A12/A17*	✓	○	××	××	××	××	○	✓	
	A02/A07*		×	××	×	○	○	○		
PHG	A11		○	×	×	○	○	○		
	A16		○	××	×	○	○	○		
	A01		○	○	×	○	○	○		
	A06		○	○	×	○	○	○		
Patient 2										
ITG	B15/B20*		×	○	×	○	○	○		
ITG/FG	B09		××	××	×	××	××	○		
	B14	✓	×	××	×	○	×	○	✓	
	B19	✓	○	××	×	○	××	○	✓	
FG	B08		××	××	○	○	××	○		
	B13		○	×	×	×	×	○		
	B18		○	×	×	○	×	○		
Patient 3										
ITG	C02	✓	×	××	×	××	××	○		
	C17		×	×	○	○	○	○		
FG	C11		○	×	×	○	×	○		
Patient 4										
ITG	D19		○	××	○	×	×	○		
	D13/D18*		×	××	××	××	××	×		✓ (D13)
	D15/D20*		○	×	○	○	○	○		
FG	D02		○	××	○	○	×	○		
	D03/D08*		×	××	×	××	×	×		✓ (D08)
	D07		×	–	××	××	–	×		
	D12	✓	××	××	×	○	××	○		
	D16		××	××	××	○	×	○		
	D17		×	××	○	○	×	○		
PHG	D01		○	○	×	○	○	○		
Patient 5										
ITG	E04		○	××	○	○	○	○		
	E08		×	××	×	××	××	○		
	E09		○	××	○	○	○	○		
	E13	✓	×	××	○	××	××	×		✓
	E18		×	××	○	××	××	×		✓
FG	E12		×	××	××	○	××	○		
	E16		○	××	○	××	××	○		
	E17		○	××	○	○	××	○		

Note: Stimulation was given in a monopolar fashion except for * that denotes bipolar stimulation.

○, no impairment; ×, slowing; ××, arrest; –, inconclusive due to afterdischarges; ITG, inferior temporal gyrus; FG, fusiform gyrus; PHG, parahippocampal gyrus; ITG/FG, border of ITG and FG.

##### Paragraph reading

The patients were asked to read aloud of a part of children's story (80–120 kana and kanji mixed words).

##### Picture naming

The patients were asked to name of 6 familiar animate and 6 nonanimate objects. The 6 items in each category were printed in black on a white card and were shown simultaneously.

##### Reading kanji/kana words

Written names of the 6 nonanimate items used for picture naming were presented for reading aloud either in kanji or kana scripts (on separate trials). All 6 words were written in black on a white card and were presented to the patients to read. Kanji is a semi-opaque orthography, whereas kana has a direct one-to-one correspondence to its phonemic form (except for the unmarked pitch accent). Consequently, both types of reading require visual decoding and speech production, but kana reading requires much less lexical-semantic support to generate the correct pronunciation (though in this test, some semantic involvement might arise given that the kana words relate to concrete object names).

##### Spoken verbal command

The patients were asked to make gestures following a simple spoken sentence (e.g., “open your mouth”).

##### Spoken word-picture matching

From its spoken name, patients were asked to touch the target picture from 6 line drawings, all selected from the same animate/nonliving category.

##### Written word-picture matching

The target was probed with a written name in the center of the panel. Ten colored pictures (1 target and 9 foils) from the same category were surrounding the written probe. This test was conducted just for Patients 1 and 2 while stimulating the core ROI (see below for definition).

##### Identification of the core ROI

The core semantic/language ROI within the vATL was defined as the cortical region, which exhibited both robust LFPs for naming (Investigation 1) and marked language impairment by stimulation. Regarding the definition of a robust LFP, N200 and N400/P400 have been reported to be language-specific (Halgren et al. 1994; Nobre et al. 1994; Nobre and McCarthy 1995). In addition, language-related LFP signals with a peak latency of approximately 300 ms have also been reported for Kanji reading (Usui et al. 2009). Therefore, we defined a robust LFP as one showing the largest amplitude in the corresponding peak. As for the criteria for electrode selection by stimulation, we assessed the electrodes to be within the core ROI when multiple expressive and receptive tasks (≥3) were impaired and, at least in one of the tasks, performance was arrested by stimulation. The ictal onset zone did not overlap with the core ROI in any of the patients (Table 1).

#### Investigation 3 (Graded Stimulation with a Semantic Battery)

##### Aims

Rather than probing semantic and language performance on many tasks across a large number of grid locations, by using a limited number of trials per stimulation site, with clinically maximal stimulation (as per clinical mapping—cf. Investigation 2), Investigation 3 focused on the semantic–language ROI (see above) and utilized a much greater number of trials to establish the relationship between graded levels of stimulation intensity and performance on an expressive (picture naming) and a receptive (written word-picture matching) semantic task. In a new approach, by using graded stimulation over a single target location, we were able to vary the intensity from 0 (sham) to 9 mA to investigate the finer, graded relationship between stimulation and impaired function. Both reaction time (RT) and accuracy were measured, given that RT can be more sensitive to the presence of mild impairments (Lambon Ralph et al. 2012). The motivation and form of this investigation was modeled on a previous study of SD patients, which established the relationship between disease severity and resultant performance on naming versus word-picture matching (Lambon Ralph et al. 2001). Specifically, this study found that both tasks declined with increasing disease severity, but that naming was much more profoundly impaired early and throughout the span of the disorder—indicating that naming is very sensitive to the presence of even mild semantic impairments. This investigation, therefore, had 2 aims: (1) to assess the hypothesis that the selected ROI in the vATL was primarily semantic in nature and (2) to establish a new graded stimulation methodology for use in this and future studies, whereby the relative performance on different cognitive tasks, are compared across different stimulation intensities—with the expectation that more sensitive tasks (in this example, naming) might require less stimulation to generate impairment (as measured by RTs and accuracy).

##### Materials and tasks

Before surgery and the later stimulation investigations (∼10 days before), we confirmed the patients' normal semantic ability on the 2 assessments and used the data to form the baseline for the later stimulation study. The patients were tested on naming of 80 colored pictures (≥90% accurate) and on a 10-alternative (within category) written word-picture matching task comprising the same 80 target items (≥95% accurate). Both assessments were Japanese versions modeled on the Cambridge 64-item semantic battery test (Bozeat et al. 2000). Based on the patients' results, 64 correct items were selected for each patient and were divided into 4 sets (each contains 16 items) matched for accuracy (100%), RT (see the open-marker lines in Fig. 3*A*), and psycholinguistic variables (word frequency/imageability/familiarity; Amano and Kondo 1999, 2000; Sakuma et al. 2005). Each set was allocated to 1 of the 4 stimulation intensity conditions [0 (sham), 3, 6, or 9 mA]. In both naming and matching, the same 16 items were probed at the same intensity condition. Item presentation was randomized within each block.

##### Stimulation site

**Figure 2.**
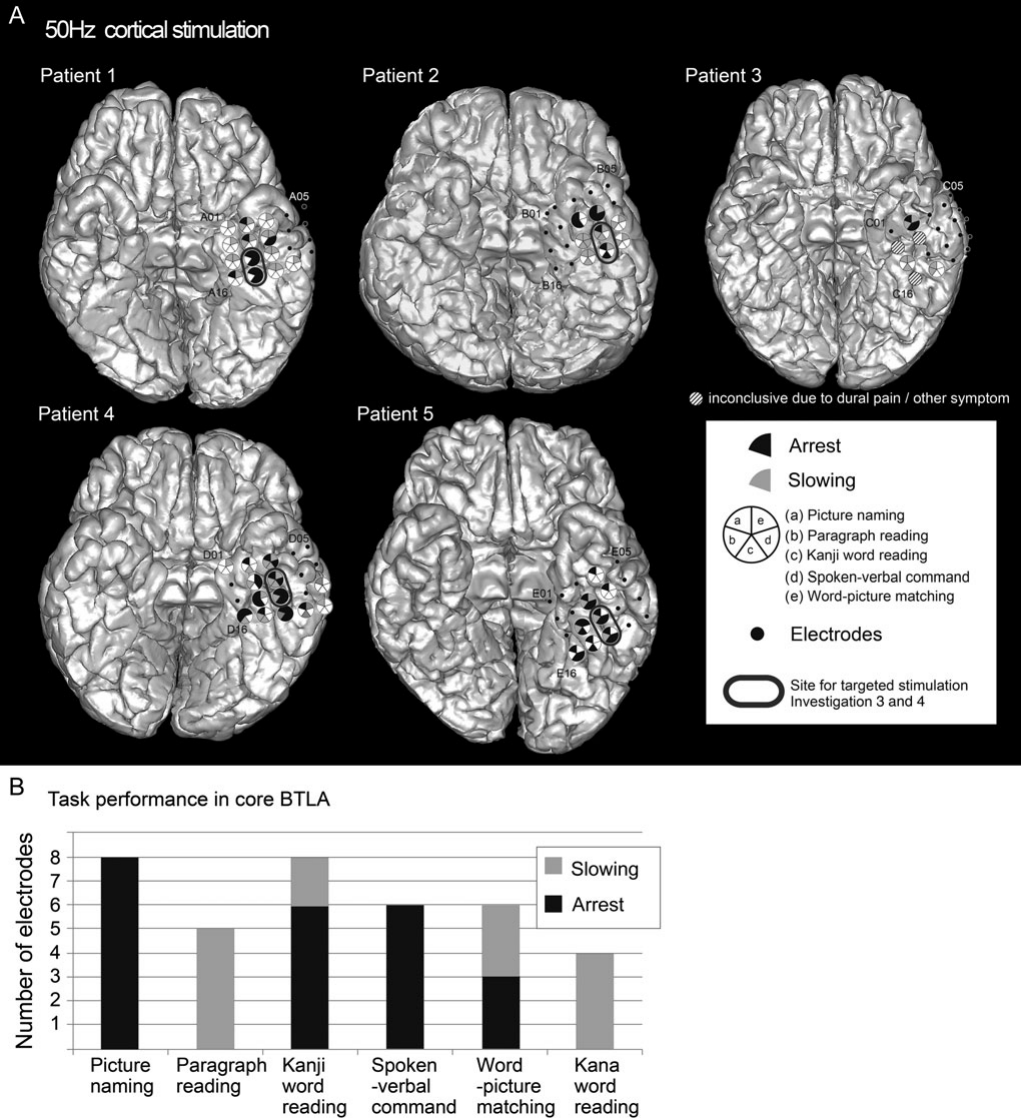
(*A*) The results of language mapping with conventional 50 Hz cortical stimulation of vATL sites in Patients 1–5. Small black dots represent subdural electrodes investigated with cortical stimulation. Electrodes showing any language impairment are illustrated with large circles with 5 segments (one for each task). Filled segments denote impairment of the corresponding task by stimulation. A black fill denotes arrest by stimulation and gray fill denotes slowing or errors. The gray ovals mark the core language electrodes used as the site for the targeted stimulation Investigations 3 and 4 in these 3 patients. In this target area, high-frequency stimulation generated impairments across all the semantically related tasks (both receptive and expressive), but spared kana word reading (which requires visual processing and speech production but not access to word meaning, unlike the other tasks), consistent with the conclusion that this region is crucial for pan-modal semantic processing. Clinical stimulation of vATL in Patients 3 and 6 was partial, inconclusive and had to be discontinued due to dural pain or other symptoms. For clarity, only the subdural grid (4 × 5 electrodes) in the basal temporal area is shown. (*B*) The performance status for each task during vATL stimulation (Patients 1, 2, 4, and 5). Picture naming, kanji word reading, and spoken verbal command task were consistently impaired upon stimulation while kana word reading was rarely impaired.

The electrode pair in the core semantic–language ROI (see above) was selected. It was A12/A17 in Patient 1 and B14/B19 in Patient 2. In Patient 2, another core ROI was identified at a more medial position (B13/B18, Fig. 2). We selected the lateral pair (B14/B19) because a pilot study revealed frequent afterdischarges upon stimulation of the medial pair.

##### Stimulation intensity

Cortical stimulation was delivered time-locked to the picture naming or written word-picture matching task with graded intensities. For each task, we conducted 4 sessions with varying intensities: 0 (baseline), 3, 6, and 9 mA (16 items, respectively).

##### Flow of a trial

**Figure 3.**
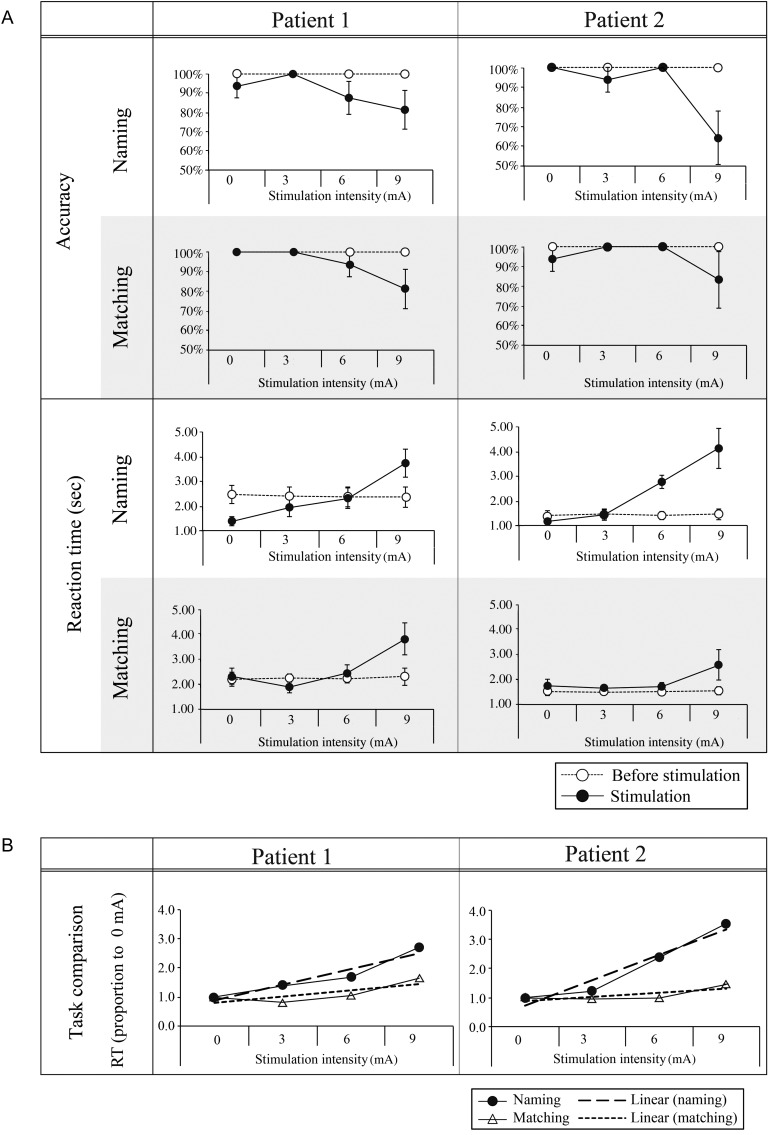
Effect of varying anterior fusiform stimulation intensity on naming and written word-picture matching performance. (*A*) The direct comparison of performance before and after stimulation for the 2 tasks in Patients 1 and 2. The same pattern was observed in both RT and accuracy, though RT was more sensitive overall to the effect of milder stimulation intensities (see the main text). As expected, performance declined (slower RTs and lower accuracy) as stimulation intensity increased. (*B*) A direct comparison (by normalization to the sham condition—see Experimental Procedures) of the relative effect of stimulation intensity on the 2 tasks for both patients. As expected, stimulation had a relatively greater impact on naming than word-picture matching (see the main text).

In both tasks, each trial started with a fixation period of 2 s, followed by display of the material (one picture for naming; a written probe surrounded by 10 pictures for matching) for 8 s, and thus an intertrial interval of 10 s. In the naming task, the patient was asked to name the picture aloud as soon as possible. In the matching task, the patient was asked to say the number shown next to the target picture. A 50-Hz electrical stimulation was given for 3 s from 1 s before the display of the material (i.e., during the latter half of the fixation period) to 2 s after the display of the material. Time-locked stimulation was given for intervention in each trial, that is, every 10 s to give intervention to all trials. First, we performed graded stimulation to the picture naming task at 4 levels of intensity in the ascending order (0, 3, 6, and 9 mA). After 30 min break, we conducted the word-picture matching task in the same ascending stimulation order. Following the standard instructions for the Cambridge Semantic Battery, we conducted naming before word-picture matching so as to minimize any cueing/priming effects on naming (though, of course, the reverse possibility of naming priming word-picture matching is possible). In all sessions, ECoG was carefully examined to monitor possible afterdischarges induced by the 3-s stimulation. All sessions were video-recorded and the verbal responses were recorded with a microphone that was placed in front of each patient. The RT for correct trials (without afterdischarges) was submitted to the analysis. Task performance was assessed by accuracy and RT for each stimulation intensity (Fig. 3).

#### Investigation 4 (Synonym Judgment and Naming vs. Number Judgment: LFP and Stimulation)

##### Aims

Patients 4 and 5 also undertook an additional investigation which directly compared LFPs and stimulation results for 2 semantic assessments (synonym judgment and naming) and a difficulty(RT)-matched number judgment task [which has been used in previous rTMS studies, fMRI experiments, and neuropsychological investigations to test/control for any nonspecific effects due to task difficulty (Pobric et al. 2007; Hoffman et al. 2010)]. The aims of this investigation included: (1) to add further confirmation of the semantic nature of the anterior fusiform–ITG ROI using both LFP and stimulation methodologies; (2) to replicate the results from naming and word-picture matching (from Investigation 3) with a different semantic task (synonym judgment); and (3) to test whether this region is crucial for both concrete and abstract concepts (i.e., is a pan-category semantic region) as suggested by recent fMRI and neuropsychological investigations (Jefferies et al. 2009; Binney et al. 2010; Lambon Ralph et al. 2012).

##### Local field potentials

Naming LFPs were collected using the same methods and materials as summarized in Investigation 1. Patient 4 and 5's naming LFPs are reported under Investigation 1 (Fig. 1). LFPs for synonym and number judgments were collected using a similar methodology. We collected 153 items for synonym judgment and number judgment tasks. Accuracy in neurologically intact participants was >96% for these items. Synonym judgment was split into 3 conditions varying the type of word (51 high-frequency concrete words [mean frequency = 18 267; mean imageability = 4.69], 51 low-frequency concrete words [mean frequency = 1897; mean imageability = 4.33], and 51 abstract/low imageability words [mean frequency = 2035; mean imageability = 3.24]). The 102 concrete items were derived from the Kanji-reading materials developed by Fushimi et al. (2009). The same number of abstract trials was added by selecting a set of low concreteness/imageability words, matched to the low-frequency set (Sakuma et al. 2005). All targets/foils were 2-character kanji compound words. The items of number judgment were derived from previous neuropsychological/rTMS studies (Pobric et al. 2007; Hoffman et al. 2010). Each trial contained a probe word/number and 3 choices, and the participant is asked to select the item that most closely matches the meaning/value of the probe.

Stimuli were presented on the PC screen every 5 s. The participants were asked to respond to the stimuli by pressing 1 of 3 designated buttons. One session consisted of 51 trials and 6 sessions (3 synonym and 3 number judgment tasks) were performed alternately. The recording method and parameters for ECoG were the same as in Investigation 1 (naming) with a band-pass filter of 0.016–300 Hz and a sampling rate of 1000 Hz. The method of offline averaging of ECoG was the same as naming LFP.

##### Stimulation

A similar methodology and approach to Investigation 3 was used in this study. Prior to surgery, Patient 4 completed the synonym and number judgment tasks as well as picture naming (the same items as Investigation 3). From these baseline assessments, we selected 60 correct trials for synonym judgment, 20 trials for number judgment, and 20 trials for naming during concurrent stimulation. Synonym judgment was split into 3 conditions (20 items for each cell) varying the type of word (20 high-frequency concrete words, 20 low-frequency concrete words, and 20 abstract/low imageability words) but matching decision times. Trials for the number judgment task were selected so that the baseline decision times were equated to the synonym task. Baseline naming times were much faster than the synonym and number judgment tasks, and thus naming trials could not be matched by RT.

**Figure 4.**
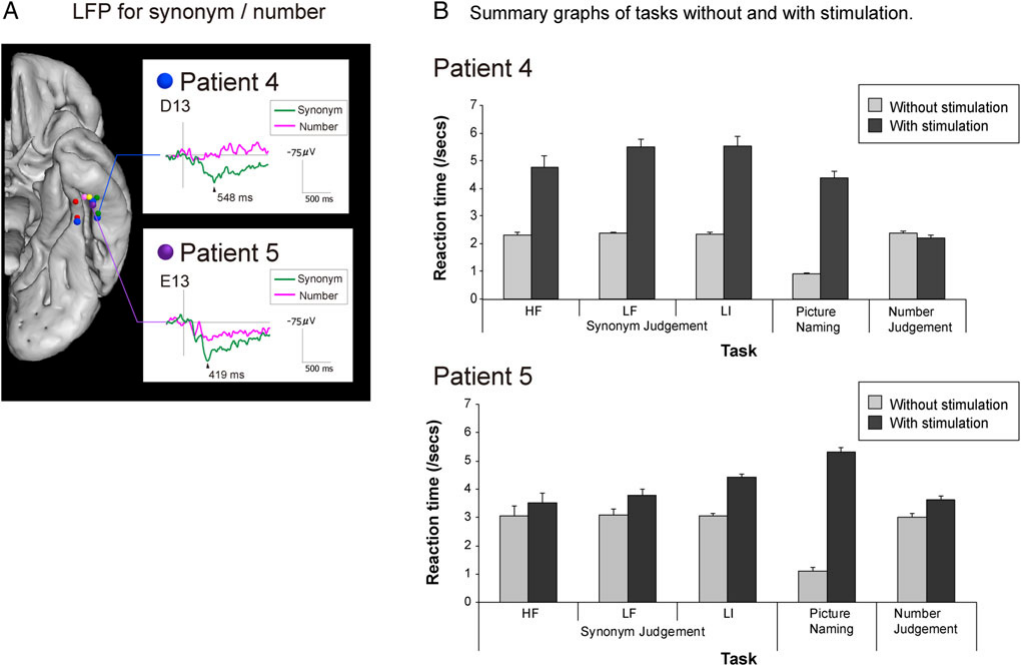
LFPs and results of direct cortical stimulation on synonym judgment, naming, and number judgment in Patient 4 and 5. (*A*) Representative LFP waveforms (average waveforms from the first and second halves of the synonym judgment session to show the stability of the data) taken from the same vATL region as that used for the direct stimulation investigation (Patient 4—electrodes D13; Patient 5—electrodes E13) and aligning with the peaks from the naming LFP studies (Fig. 1). (*B*) Both patients' RT data across the 3 tasks with and without concurrent (7 mA/3 s) stimulation. Synonym decision times were substantially and significantly slowed for all 3 types of word [high-frequency concrete (HF), low-frequency concrete (LF), and low imageability/abstract (LI)]. Accuracy was also substantially reduced (see the main text). As found in Investigation 3 for Patients 1 and 2 (Fig. 3), stimulation at this same site also generated significant and considerable slowing of picture naming. In contrast, however, neither speed nor accuracy of difficulty (RT)-matched number judgments were affected, suggesting a semantically selective effect of stimulation at this site.

For Patient 4, electrodes D08/D13 were identified as the core semantic–language ROI (see above). For Patient 5, electrodes E13 were identified as the core semantic–language ROI (stimulation was delivered to the pair of electrodes E13/E18). Stimulation was delivered time-locked to the onset of the task presentation for 3 s thereafter. A fixed intensity (50 Hz, 7 mA) was used. Afterdischarge was not induced. RTs, accuracy, and errors were recorded for later evaluation and analysis. The results are summarized in Figure 4.

#### Anatomical Localization of Subdural Electrodes in Individual and Standard Space

A magnetization-prepared rapid gradient-echo (MPRAGE) sequence was applied for anatomical *T*_1_-weighted volume data acquisition. MPRAGE volumetric scan was performed before and after implantation of subdural electrodes as a part of presurgical evaluations. In the volumetric scan taken after implantation, the location of each electrode was identified on the 2D slices using its signal void due to the property of platinum alloy (Matsumoto et al. 2004). To compare the findings obtained in individual patients with previous fMRI/PET semantic studies (the results of which are reported in standard space), the location of electrodes was coregistered to the presurgical 3D-MRI, and then normalized to the MNI standard space for anatomical localization. Electrodes identified on the *T*_1_ volume acquisition (1.5 T, MPRAGE) taken after grid implantation were non-linearly coregistered to the *T*_1_ volume acquisition taken before implantation (3T, MPRAGE), then to the MNI standard space (ICBM-152) using FNIRT of the FSL software (www.fmrib.ox.ac.uk/fsl/fnirt/). This method has been reported elsewhere for standardization of the electrode locations (Matsumoto et al. 2012).

## Results

### Investigation 1 (LFPs)

In all 6 patients, LFPs were recorded during the semantic task of picture naming. Robust responses (Supplementary Fig. 1 for the full LFP maps) were observed at and around the left (Patients 1–5) and right (Patient 6) anterior fusiform gyri (3.8–5.2 cm from the temporal pole in the MNI space). A negative activity with a peak latency of 230–300 ms was observed in 2 patients (1 and 3), followed by a positive/negative activity peaking at 310–450 ms in 5 cases (1–5). Patient 6 showed a negative activity with peak latency around 300 ms, similar to left cases. Figure 1*A* shows the LFP for each patient at this site. It is striking that this location is almost exactly the same as the site of peak activation observed for a range of different verbal and nonverbal semantic tasks using distortion-corrected fMRI or PET in neurologically intact participants (Fig. 1*C*)—indicating a strong convergence of evidence across LFP, fMRI, and PET. In the subsequent investigations, therefore, the necessity of this region (Fig. 1*B*) was tested and confirmed in a series of direct stimulation experiments.

### Investigation 2 (Conventional Functional Cortical Mapping)

We performed conventional presurgical evaluations using high frequency, approximately 10 mA electrical cortical stimulation with standard clinical materials (Matsumoto et al. 2011). By limiting the number of trials per stimulation location, it is possible to map the response characteristics of all grid positions across 6 expressive/receptive and auditory/visual language tasks (see Experimental Procedure). As summarized in Figure 2 and Table 2, high-frequency stimulation of the anterior fusiform/ITG (sites A02/07/08/12/17 for Patient 1; B09/14/19 for Patient 2; C02 for Patient 3; D03/07/08/12/13/16/18 for Patient 4; and E08/12/13/16/18 for Patient 5), but not other positions, generated impairments in the semantically related tasks (both receptive and expressive). Kana word reading was spared in Patients 1–3. Patients 4 and 5 showed slight slowing (not arrest) of kana word reading but only in 3 of the 13 electrodes in Patient 4 and in 2 of the 8 electrodes in Patient 5 which generated semantic impairment. Clinical stimulation of the vATL in Patients 3 and 6 had to be discontinued due to dural pain or other symptoms. To clarify the features of the target region, Figure 2*B* shows the incidence of impaired task performance during stimulation. Picture naming, which is very sensitive to semantic status, was impaired in all of the core BTLA electrodes upon stimulation. Overall, these results indicate that the vATL is neither a language- nor visually specific region, but is crucial for semantic processing, irrespective of the sensory input modality.

### Investigation 3 (Graded Stimulation with a Semantic Battery)

For 2 patients (1 and 2), the precise function of the same anterior fusiform–ITG region was more thoroughly investigated by using a novel graded stimulation protocol with expressive (picture naming) and receptive semantic tasks (written word-picture matching—changing the input modality from the spoken version used in Investigation 2 and thus providing a secondary test of the multimodal character of this region). As can be seen in Figure 3, there was a clear effect of anterior fusiform–ITG stimulation on both tasks. Polyserial correlation showed a significant relationship between (slowed) RT and increasing stimulation intensity on both tasks (Figure 3*A*; polyserial correlation coefficients >0.272, *P* < 0.05). At the strongest intensity level, RT was significantly slower than that before stimulation (*P* < 0.05). Accuracy also declined at this stimulation intensity. Additionally, this milder stimulation method evoked errors of commission as well as omission in Patient 1. All errors were semantically closely related (e.g., racoon→ “koala”; sheep→ “deer”), suggesting that the nature of the stimulation-induced impairment was semantic, like that found in SD patients. Patient 2's errors were dominated by omissions (the most common naming error type in SD).

Finally, we compared the relative effect of graded stimulation on naming [slope for the by-item regression line in Fig. 3 = 0.17 (SE = 0.03) for Patient 1 and 0.27 (SE = 0.03) for Patient 2] versus word-picture matching [slope = 0.06 (SE = 0.02) for Patient 1 and 0.04 (SE = 0.02) for Patient 2]. As can be observed in Figure 3*B*, for both patients, the effect on naming was significantly greater than that on word-picture matching (asymptote test on the slope values: *P* < 0.01 for both patients). This outcome directly mirrors SD patients' rapid decline in naming accuracy compared with word-picture matching as the disease progresses (Lambon Ralph et al. 2001) and also the comparison of weak (leading to anomia) versus intense (global aphasia) stimulation of the BTLA conducted by Lüders et al. (1991). Crucially, given that there were more pictures for the visual system to process in matching than naming, this pattern of results excludes a purely higher-order visual processing account for the function of this area.

### Investigation 4 (Synonym Judgment and Naming vs. Number Judgment: LFP and Stimulation)

Previous distortion-corrected fMRI studies have suggested that the anterior fusiform/ITG is important for a wide variety of semantic tasks, including the processing of concrete and abstract concepts (Binney et al. 2010; Visser and Lambon Ralph 2011). Lateral ATL regions have been similarly implicated and their necessity confirmed through rTMS studies (Pobric et al. 2007). The assessments from these previous studies (naming, synonym judgment, and a nonsemantic, difficulty-matched number judgment task) were utilized in a new grid electrode investigation of the anterior fusiform–ITG with Patients 4 and 5. Unlike other neuroscience techniques, subdural electrode grids permit both functional mapping (LFP) and stimulation in the same participant. Accordingly, Patients 4 and 5 completed the tasks under normal conditions, while LFPs were measured, and then again during concurrent stimulation of the anterior fusiform–ITG.

Like Patients 1–3, robust LFPs were observed during naming in the anterior fusiform–ITG for Patients 4 and 5 (Fig. 1). A similar LFP map was obtained for the synonym judgment task. Figure 4A shows averaged LFP waveforms for the anterior fusiform–ITG location (electrodes D13 in Patient 4 and E13 in Patient 5—at which stimulation was later delivered) with a significant positive peak at 400–550 ms (Supplementary Fig. 2 for the full synonym judgment LFP maps). In comparison, the nonsemantic number judgment task did not generate any reliable LFPs in this area, indicating that processing in the region is semantically selective. The results of direct stimulation mirrored the LFP findings. The analysis of Patient 4’s RT data revealed a significant stimulation × task interaction (*F*_2,71_ = 63.5, *P* < 0.001). This pattern (Fig. 4*B*) reflected the fact that 7 mA/3 s concurrent stimulation of the anterior fusiform–ITG significantly and substantially slowed synonym judgments for all word types [*t*_(38)_ = 13.6, *P* < 0.001] and naming responses [*t*_(14)_ = 14.2, *P* < 0.001], but had no effect on number judgments [which showed a non-significant trend toward faster decision times: *t*_(19)_ = -1.47, *P* = 0.15]. On the more demanding synonym judgment task, concurrent stimulation also reduced Patient 4's accuracy from 100% at baseline to 65% (McNemar *P* < 0.001). As found in Investigation 3, the lower level of stimulation allowed generation of a semantically related naming error (penguin→ “pelican”), again underlining the notion that this region is crucial for semantic processing. There was no change in Patient 4's accuracy on the number judgment tasks. A very similar result was obtained for Patient 5. There was a significant stimulation × task interaction in response times (*F*_2,50_ = 76.2, *P* < 0.001). Again, this interaction reflected a substantial and significant slowing of RTs for synonym judgments [*t*_(28)_ = 5.86, *P* < 0.001] and naming [*t*_(7)_ = 27.8, *P* < 0.001], but a much smaller effect on number judgments [*t*_(15)_ = 3.4, *P* < 0.004]. In comparison with Patient 4, Patient 5's accuracy on the naming and synonym judgment tasks was much more strongly suppressed by stimulation (naming from 100% to 40%: McNemar *P* < 0.001; synonyms from 100% to 48%: McNemar *P* = 0.13). All of the Patient 5's stimulation-induced naming errors were omissions. In comparison with the 2 semantic tasks, a small drop in accuracy in the number judgment task was not significant (McNemar *P* = 0.13). In summary, the stimulation and LFP methods produced highly convergent findings, namely that the anterior fusiform/ITG is a semantically selective region.

## Discussion

Semantic memory is a crucial cognitive function that brings meaning to our verbal and nonverbal experience, and generalizes this knowledge across different contexts and time (Rogers et al. 2004; Patterson et al. 2007; Lambon Ralph et al. 2010; Lambon Ralph 2014). The contribution of the vATL to semantic memory was explored through a systematic series of investigations that utilized subdural electrode grids, implanted in 6 patients as part of the presurgical evaluation of their partial epilepsy or brain tumor. Such electrode grids provide the rare yet dual opportunities, in the same participants, to evaluate the contribution of the cortical area immediately below each electrode site to the cognitive task of interest and to confirm the necessity of the functionally identified regions through direct stimulation, leading to transient disruption of processing at the cortical site. A strikingly consistent neuroanatomical and functional picture emerged from these investigations. LFP maps identified the anterior fusiform–ITG as a core region in both expressive (picture naming) and receptive semantic tasks (synonym judgments), but not in nonsemantic, difficulty-matched assessments (number judgments). The potential importance of this region to semantic processing was emphasized in that stimulation of this area led to transiently impaired performance across a range of semantic tasks with both visual and auditory inputs (picture naming, spoken and written word-picture matching, synonym judgments, and comprehension of verbal commands) or tasks that require semantic support (paragraph reading and kanji word reading), but left performance on the difficulty-matched number task unaffected. Although only demonstrated in a limited number of cases (due to the rarity of this clinical procedure), these results directly parallel those found in distortion-corrected fMRI and other methods (see below). These consistent cross-modality results indicate that the vATL is not a higher-order visually specific region (Kravitz et al. 2013). Instead, there is a shift from visual processing in ventral occipitotemporal areas to more modality-general (semantic) representations in the vATL (Visser and Lambon Ralph 2011; Peelen and Caramazza 2012; Visser et al. 2012). Our data both replicate and extend the seminal stimulation and cortical-evoked potential studies (Lüders et al. 1986, 1991; Nobre et al. 1994) in that, like Lüders et al, we found stimulation of the region generated language-related deficits but also, in keeping with Nobre et al., we found that the stimulation effects and cortical-evoked responses were associated with semantic representation. Indeed, although Lüders et al (1991) argued that the ATL region might be a purely language-related area (on the basis that mild stimulation generated anomia and intense stimulation led to global aphasia), the combination of data from this study with the selective semantic impairment observed throughout the course of SD (with preservation of phonological and syntactical aspects of language) and recent fMRI explorations (Visser and Lambon Ralph 2011; Peelen and Caramazza 2012) suggests that the function of this region may well be centered on verbal and nonverbal aspects of semantic memory.

When compared in the MNI standard space, the location of the core vATL region in the 6 patients corresponded closely with the activation peaks identified in distortion-corrected fMRI and H_2_O-PET activation studies of multimodal semantic processing (Fig. 1). In addition to the temporal pole, this same ventral region is found to have greater atrophy than other ATL sites in SD (Galton et al. 2001), and SD patients' level of remaining semantic performance is correlated with the degree of glucose hypometabolism in this same region (Mion et al. 2010). The importance of the anterior fusiform–ITG for semantic processing is also supported by previous reports of semantic and category-related evoked responses in studies of written word and picture recognition (Nobre et al. 1994; Chan et al. 2011). The current results also fit very closely with recent distortion-corrected fMRI investigations that have identified pan-modality semantically related ventrolateral temporal activations, centered on the anterior fusiform–ITG (Binney et al. 2010; Visser and Lambon Ralph 2011). Although the data were limited by clinical restrictions, the current study also found evidence to suggest that semantic processing may be supported by the anterior fusiform–ITG region, bilaterally. Patient 6 exhibited LFP for picture naming in homologous right anterior ventral regions as those observed in the left for Patients 1–5 (Fig. 1). This result is consistent with studies of patients with unilateral anterior temporal damage, functional neuroimaging and rTMS investigations of neurologically intact participants, as well as classical neurosurgery studies in primates and humans all of which indicate that both left and right ATL regions contribute to semantic representation (Klüver and Bucy 1937, 1939; Terzian and Dalle Ore 1955; Lambon Ralph et al. 2009, 2012; Schapiro et al. 2013).

Overall, the study's results support the hypothesis that the anterior fusiform–ITG is the center point of a graded transmodal representational ATL hub, which interacts with modality-specific sources of information coded across a distributed network of secondary association cortices (Rogers et al. 2004; Patterson et al. 2007; Pobric et al. 2010b; Lambon Ralph 2014). Such proposals fit with human and nonhuman primate connectivity studies, which demonstrate the convergence of major white-matter pathways from disparate primary sensory and motor regions into these ATL areas (Morán et al. 1987; Binney et al. 2012). By pairing modality-specific information sources with a transmodal representational hub in this way, the resultant neurocomputational system is capable, not only of fusing multimodal features together into coherent concepts but also to compute novel, semantic-based generalizations. These are core, necessary characteristics of the semantic system, the basis of which has puzzled philosophers, behavioral neurologists, and cognitive scientists alike, over many years (Rosch 1975; Smith and Medin 1981; Wittgenstein 2001; Lambon Ralph et al. 2010).

Three further issues should be considered: (1) The apparent disparity between stimulation of the vATL versus resection of it; (2) the localization of cortical stimulation; and (3) the relationship of the vATL to other regions implicated in multimodal semantic processing.

From a general clinical perspective, there appears to be a disparity between the apparently minimal neuropsychological deficits of patients with ATL resection in the chronic phase versus the semantic impairment that follows from electrical stimulation of the vATL or is found in SD. Post-surgical language/semantic impairment is much more obvious in the acute stage, but diminishes over time. Using sensitive measures, however, various studies have found evidence for remaining semantic impairment underpinning the patients' anomia even in the chronic phase (Wilkins and Moscovitch 1978; Antonucci et al. 2008; Lambon Ralph et al. 2012). The partial recovery of semantic function after unilateral ATL resection suggests that there are compensatory mechanisms which require further exploration in future research. At least one possibility (as noted above) is that the ATL regions might act in a bilateral fashion (cf. Schapiro et al. 2013), meaning that significant long-term chronic semantic impairment only follows after bilateral resection (in monkeys, primates, or one human case: Brown and Schafer 1888; Klüver and Bucy 1939; Terzian and Dalle Ore 1955) or in bilateral diseases such as SD or HSVE (Lambon Ralph et al. 2007).

A second potential issue is whether some or all of the observed deficits after vATL stimulation are due to stimulation spreading through the white-matter connections to Wernicke's area. For example, we know from single-pulse cortico-cortical connectivity studies that stimulation of the vATL generates an evoked response in Wernicke's area, and vice versa (Matsumoto et al. 2004; Koubeissi et al. 2012). This opens up the possibility that the transient impairment of semantic processing after vATL repetitive stimulation reflects a combination of inhibition/disruption not only in the stimulated region (which we know activates during semantic tasks from the LFP data) *and also* the areas to which it is connected. Indeed, a combination of local and remote stimulation could amplify the observed impairment. To test this hypothesis, future studies are required, in which grid electrodes are implanted over the vATL-connected component of Wernicke's area to test if stimulation leads to a selective semantic impairment. While we cannot rule out this possibility at this stage, we think it may be unlikely for 3 reasons. First, previous neurophysiological studies have found that current density is maximal only beneath the stimulated electrode (Nathan et al. 1993) and thus, stimulation-related effects, if present, are most likely to represent the local cortical function. Secondly, single-pulse stimulation can produce cortico-cortical-evoked potentials to remote cortices directly through white-matter pathways (Yamao et al. 2014), but it does not necessarily mean that functional impairment will occur in the remotely connected sites; recent combined stimulation fMRI investigations (Logothetis et al. 2010) indicate that direct repetitive stimulation does not propagate beyond the first synapse unless it is very high frequency (>200 Hz). Thirdly, in considering the impact of remote stimulation of Wernicke's area on semantic performance, it is important to compare the effects of damage to ATL versus Wernicke's area. Recent comparative neuropsychological studies have found that not only are different symptoms found in SD (multimodal, selective semantic impairment without generalized language and cognitive deficits) and Wernicke's aphasia (primary phonological disturbance with variable semantic and cognitive impairment), but also the nature of the semantic impairment in each group is different (Ogar et al. 2011; Robson et al. 2012).

Finally, it should be noted that the ATL is not the only cortical region that is involved in multimodal semantic processing (for recent reviews, see Binder et al. 2009; Visser et al. 2010; Lambon Ralph 2014). Other regions include inferior prefrontal cortex, anterior cingulate, posterior middle temporal gyrus, angular gyrus (AG), and the intraparietal sulcus. There is a growing consensus that some of these regions (e.g., PFC and IPS) are not implicated in semantic representation per se, but rather support multimodal executive processes that interact with semantic knowledge (Thompson-Schill et al. 1997; Noonan et al. 2013). The contribution of the remaining areas is less clear. The role of AG, for example, has been debated for many years. Considered in the context of patients with semantic aphasia, Head (1926), Goldstein (1936), and Luria (1976) concluded that it did not support semantic representations, but rather underpinned more domain general symbolic processing mechanisms which are required in semantic and nonsemantic higher cognitive tasks [see also Jefferies and Lambon Ralph (2006); Lambon Ralph (2014)]. In contrast, Geschwind (1972) proposed that the AG's anatomical position and connectivity were ideal for semantic representation. Similar contrastive hypotheses have emerged from the functional neuroimaging literatures with some researchers proposing that the AG is another representation hub (in addition to the ATL), perhaps specialized for coding event knowledge (cf. Binder and Desai 2011). Other researchers have noted that the AG is not only activated by semantic tasks but also implicated in many different cognitive domains (including episodic memory, attention, mathematical processing, etc.), and thus may be much more domain general in nature (Cabeza et al. 2012). Future studies are required to explore how representational and control areas work together to generate semantic cognition and, if there are multiple representational hubs, how semantic knowledge is distributed between them.

Before finishing we should note that as well as adopting standard clinical methods to probe the function of the anterior fusiform–ITG, the current study introduced various new clinical protocols. For example, in addition to utilizing maximal stimulation intensities to investigate which anterior temporal regions give rise to transient “semantic arrest” (the standard clinical method which probes many potential sites albeit with a very limited number of trials per stimulated area), we performed graded-intensity stimulation at a single location (the anterior fusiform–ITG) to investigate the relative effects on expressive (naming) and receptive (word-picture matching) semantic tasks. This new methodology was inspired by previous studies of the disease progression in SD patients, which have used this neurological model to establish the relationship between the degree of damage to the underlying semantic system and the resultant performance level across different tasks (Lambon Ralph et al. 2001; Woollams et al. 2007). Thus, like these previous neuropsychological investigations, the current study was able to demonstrate that increasing stimulation of the anterior fusiform–ITG generated impairments in both naming and word-picture matching, but that the effect on the expressive task of naming was relatively greater [mirroring directly the neuropsychological data of SD patients (Lambon Ralph et al. 2001)]. Utilizing these milder stimulation intensities (than those designed to cause temporary arrest of function) also led us to adopt RT measures and analysis of commission naming errors in stimulation studies for the first time. These provided additional evidence in favor of the crucial role of the anterior fusiform–ITG, but we also hope that these new methodologies can be generalized to invasive investigations of other cognitive functions.

In conclusion, the present systematic, combined stimulation and LFP study located semantic processing in and around the anterior fusiform–ITG. This neuroanatomical location is in good agreement with the activation foci for semantic processing in various functional neuroimaging studies. The measured semantic function and induced transient impairment at the anterior fusiform–ITG was found to be both multimodal (e.g., elicited by auditory or visual inputs) and pan-category (e.g., for concrete and abstract concepts), and graded stimulation of the region elicited a similar form of semantic impairment as observed across the disease progression of SD. Taking these findings together, the anterior fusiform–ITG appears to be critically important for semantic processing. A future prospective neuropsychological study of patients who need additional resection of the vATL during subtemporal hippocampectomy, which utilizes advanced neurosurgical techniques to avoid resection of the anterior ventral temporal surface and the temporal stem (Mikuni et al. 2006), is needed to clarify the functional reorganization of the semantic network after the removal of this critical area.

## Supplementary material

Supplementary material can be found at http://www.cercor.oxfordjournals.org/.

## Funding

This research was supported by an MRC-UK (MR/J004146/1) program grant (MALR), grants-in-aid for Scientific Research (B) 26282218 (R.M.) and 26293209 (A.I.), grant-in-aid for Exploratory Research 26560465 (R.M.) from the Ministry of Education, Culture, Sports, Science and Technology of Japan (MEXT), grants-in-aid for Research on Measures for Intractable Diseases (H26-Nanjii-Ippan-051) (A.I.) from the Japanese Ministry of Health, Labor and Welfare, and SPIRITS (Supporting Program for Interaction-based Initiative Team Studies) (R.M.) from Kyoto University. Funding to pay the Open Access publication charges for this article was provided by an RCUK block grant to the University of Manchester.

## Supplementary Material

Supplementary Data
