# Supplementary material for: Direct Exploration of the Role of the Ventral Anterior Temporal Lobe in Semantic Memory: Cortical Stimulation and Local Field Potential Evidence From Subdural Grid Electrodes
Source: Cereb Cortex. 2014 Dec 9;25(10):3802–17. doi: 10.1093/cercor/bhu262 (PMC4585516; doi:10.1093/cercor/bhu262)
Supplement: Supplementary Data [file supp_25_10_3802__index.html]

Direct Exploration of the Role of the Ventral Anterior Temporal Lobe in Semantic Memory: Cortical Stimulation and Local Field Potential Evidence From Subdural Grid Electrodes — Supplementary Data 

# Direct Exploration of the Role of the Ventral Anterior Temporal Lobe in Semantic Memory: Cortical Stimulation and Local Field Potential Evidence From Subdural Grid Electrodes

## Supplementary Data

Supplementary Data

**Files in this Data Supplement:**

- Supplementary Figure 1 - jpg file
- Supplementary Figure 2 - jpg file
- Supplementary Table 1 - xls file
- Supplementary Table 2 - xls file
