# Supplementary figures and images for: Direct Exploration of the Role of the Ventral Anterior Temporal Lobe in Semantic Memory: Cortical Stimulation and Local Field Potential Evidence From Subdural Grid Electrodes
Source: Cereb Cortex. 2014 Dec 9;25(10):3802–17. doi: 10.1093/cercor/bhu262 (PMC4585516; doi:10.1093/cercor/bhu262)

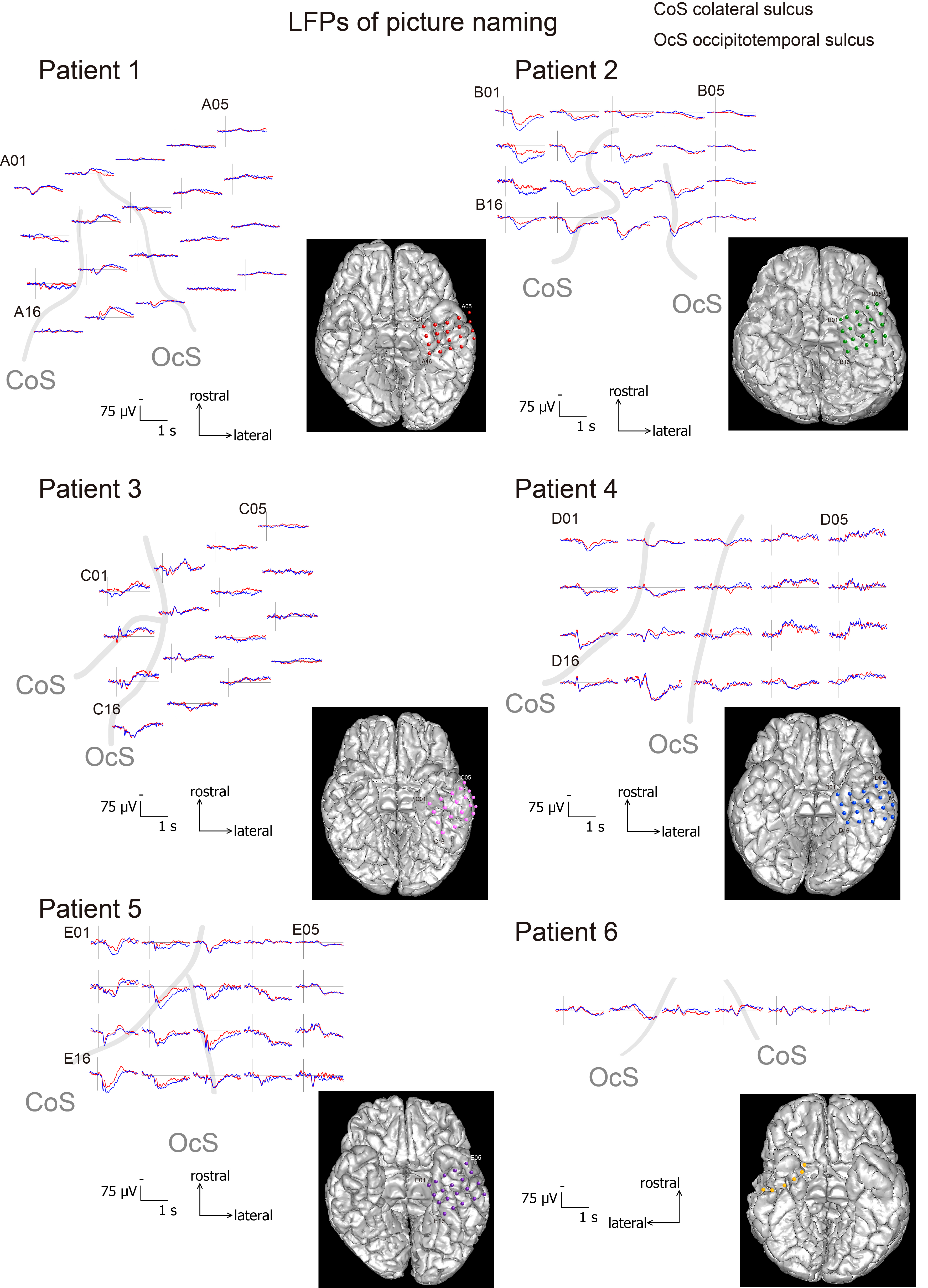

Supplement: Supplementary Data [file supp_bhu262_bhu262supp_fig1.jpg]

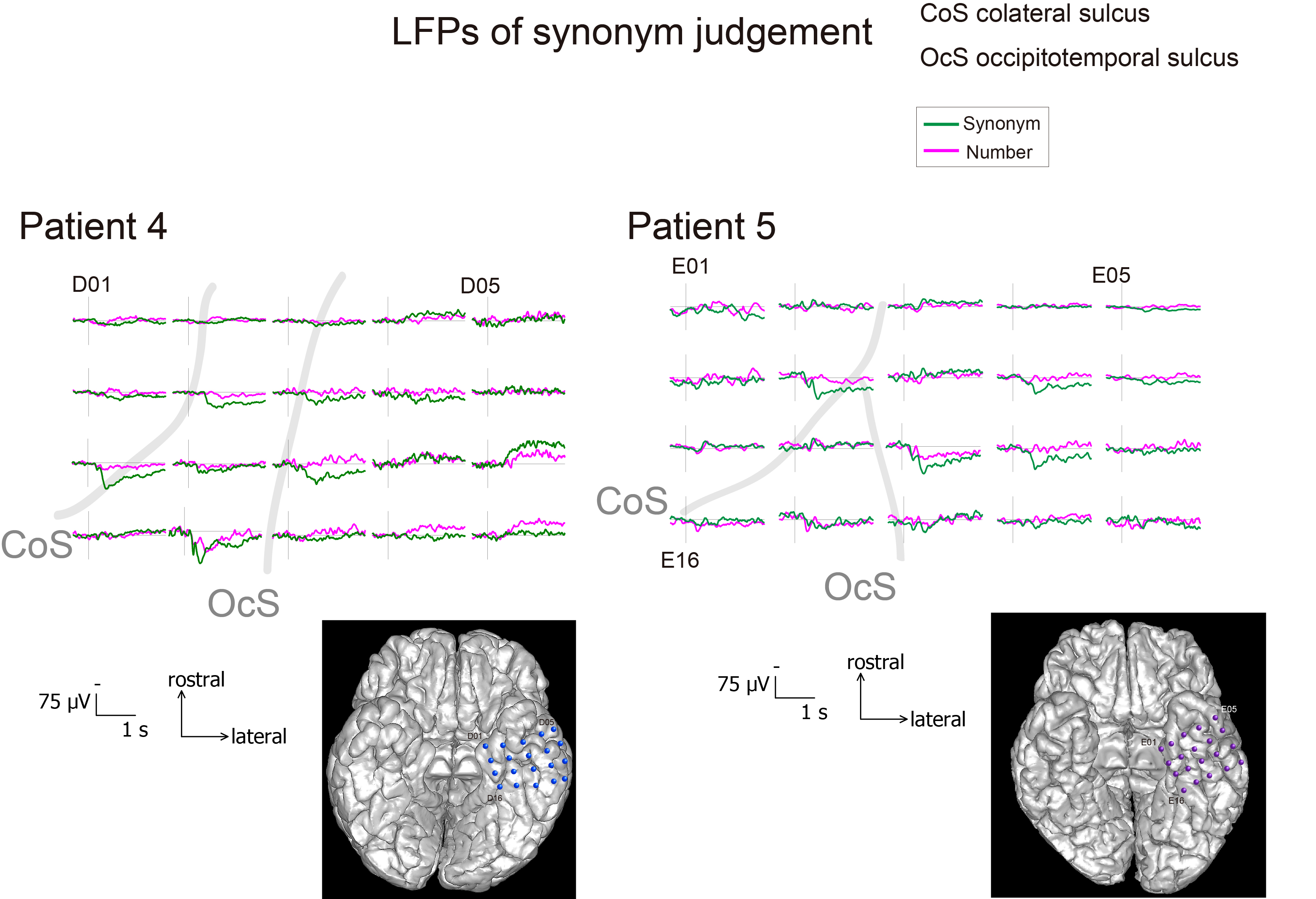

Supplement: Supplementary Data [file supp_bhu262_bhu262supp_fig2.jpg]
